# Irrational Beliefs

**DOI:** 10.5964/ejop.v15i1.1903

**Published:** 2019-02-28

**Authors:** Iris Žeželj, Ljiljana B. Lazarević

**Affiliations:** a Department of Psychology, Laboratory for Social Psychology, Faculty of Philosophy, University of Belgrade, Belgrade, Serbia; b Institute of Psychology and Laboratory for the Study of Individual Differences, Faculty of Philosophy, University of Belgrade, Belgrade, Serbia

**Keywords:** irrational beliefs, cognitive biases, intuitive thinking, epistemologically suspect beliefs, paranormal beliefs, conspiracy theories

## Abstract

Irrational beliefs are often used as an umbrella term that comprises a variety of psychological constructs: from specific cognitive biases to a wider class of epistemologically suspect beliefs (superstitions, paranormal and pseudoscientific beliefs, conspiracy theories etc.) or cognitive styles (analytical versus intuitive thinking), but also unsubstantiated self-related beliefs. This collection of papers illustrates this diversity well. Apart from the descriptive portion of the data, which has merit on its own, the authors provide important methodological innovations in the way these beliefs are measured, but also look deeper in their functionality and consequences.

No one familiar with modern empirical psychology will contest the assertion that reasoning and behavior do not always comply with rationality norms. If we label that deviations as irrational, we end up with an umbrella term that comprises a variety of psychological constructs: from an ever-growing list of specific cognitive biases to a wider class of epistemologically suspect beliefs (superstitions, paranormal and pseudoscientific beliefs, conspiracy theories etc.) or cognitive styles (analytical versus intuitive thinking), but also unsubstantiated self-related beliefs (the ones contested in varieties of cognitive-behavioral therapies). Such irrational beliefs are definitely not rare, pathological or culturally specific phenomena. On the contrary, they are widespread and cannot be tied to any specific social environment.

A non-exhaustive search using Google Scholar search engine revealed that since 2008, the term *irrational beliefs* has been mentioned in 15500 scientific papers. Irrational beliefs are explored in a range of domains and contexts, like psychotherapy (especially REBT), open- and closed-mindedness, cognitive dissonance, pseudoscience, paranormal beliefs, conspiratorial thinking, gambling, health beliefs, etc.

In this special issue, we were interested in the distribution of irrational beliefs in general and specific populations, but also in their perceptive, cognitive and motivational determinants and their individual and group-related outcomes (e.g. performance on different tasks, health behavior, civic activism, intergroup relations). Following the call, several groups of authors have contributed their most recent research on the related phenomena (Table 1).

**Table 1:** Overview of Research Studies in the Special Issue Irrational Beliefs

Authors	Type of Irrational Belief	Main Issues Examined
Teovanović	Cognitive Biases	Individual differences in susceptibility to the anchoring effect
Damnjanović, Novković, Pavlović, Ilić, & Pantelić	Cognitive Reflection Task	A reference point as a cue for rational reasoning in CRT
Damnjanović, Ilić, Pavlović, & Novković	Cognitive Biases	The impact of involvement on parental outcome bias
Petrović, Međedović, Radović, & Radetić Lovrić	Conspiracy mentality	Conspiracy mentality and its relevance for Readiness for Reconciliation
Bilewicz, Witkowska, Pantazi, Gkinopoulos, & Klein	Conspiracy Theories	The role of Conspiracy Beliefs in breaking social cohesion
Lukić, Žeželj, & Stanković	Conspiracy Theories	Proneness to endorse contradictory conspiracy beliefs
Kostovičová	Epistemically Suspect Beliefs	Fostering luck-related superstition to stimulate rationality
Branković	Extrasensory perception (ESP)	Study 1 - measurement of ESP and cognitive styles as predictors of ESP Study 2 - motivational foundations of ESP (fear of death, the locus of control)
Jokić & Purić	Cognitive styles – Rational and Experiential thinking	The role of Trait emotional intelligence in predicting Rational and Experiential thinking
Erceg, Galić, & Bubić	Epistemically Suspect Beliefs	The role of Individual differences in prediction of Epistemically Suspect Beliefs

In three papers cognitive biases are treated as a form of irrational thinking, in line with the framework in which biases are defined as a systematic pattern of deviation from norm or rationality in judgment (Haselton, Nettle, & Andrews, 2005).

**Teovanović (2019, this issue)** was interested in individual differences in anchoring effect (Tversky & Kahneman, 1974), one of the most reliable and robust cognitive biases. He focused on the extent to which cognitive abilities, cognitive reflection, and basic personality traits defined by the Big five model (Costa & McCrae, 1992), contribute to susceptibility to the anchoring effect. In addition, the author introduced a novel within-subject procedure allowing for simultaneous manipulation of experimental factors of anchor’s distance and direction, and assessment of individual differences in susceptibility to anchoring effect. Overall, anchoring bias shared a small amount of variance with cognitive abilities, cognitive reflection and personality traits. However, cognitive reflection moderated the relationship between intellectual abilities and proneness to anchoring, suggesting that engaging in the cognitive process of adjustment leads to a lower anchoring bias.

A study by **Damnjanović, Novković, Pavlović, Ilić, and Pantelić (2019, this issue)** investigated how introducing a reference point in Cognitive Reflection Task (CRT; Frederick, 2005) influences the switch between the fast, heuristic system, and the deliberate system. In a counterbalanced repeated design, the authors used two versions of a CRT, i.e. with and without a reference point. The findings of this study supported the hypothesis that introducing reference point increases performance on the CRT and lowers cognitive bias.

In the third study in this group, the authors **(Damnjanović, Ilić, Pavlović, & Novković, 2019, this issue)** explored how sensitive parents are to outcome bias depending on their involvement in the decision and the domain of decision. They presented the parents with different scenarios varying by involvement (high vs. low), and domain (health vs. non-health related). The scenarios were followed by a positive, a negative outcome or no outcome at all (control situation). Highly involving decisions yielded weaker outcome bias than low-involvement decisions in both health and non-health domain. Introducing a neutral, control group enabled them to further interpret the outcome effects - a highly-involving dilemma followed by negative outcome did not produce significantly different evaluation compared to the evaluation of a decision without an outcome. Although the results confirm the robustness of the outcome bias as a phenomenon, at the same time they illustrate the conditions that could diminish or eliminate it.

Three papers dealt with conspiratorial beliefs. Specifically, two papers explored the consequences of conspiracy beliefs on important societal outcomes, while one paper investigated the inherent structure of conspiratorial beliefs. A study by **Petrović, Međedović, Radović, and Radetić Lovrić (2019, this issue)** shed new light on the psychological roots of unwillingness to reconcile. In addition to Ethos of Conflict (Bar-Tal, 2007) and basic social attitudes (Saucier, 2013), which are already found to be important ingredients in unwillingness to reconcile, the authors introduce the Conspiracy Mentality, i.e. the proneness to conspiracy beliefs (Bruder et al., 2013), as an important factor hindering reconciliation and facilitating prolongation of intergroup conflict. Their findings suggest that Conspiracy Mentality mediates the relationship between basic social attitudes and Ethos of Conflict, which impose the necessity of taking it into consideration when developing policies and programs aimed to facilitate reconciliation in conflict zones.

Following a similar line of thought, **Bilewicz, Witkowska, Pantazi, Gkinopoulos, and Klein (2019, this issue)** looked into how conspiratorial thinking in response to collective traumas can lead to societal divides and the breakdown of social cohesion. Drawing from a nationally representative sample of Polish citizens, they compared the ones who endorsed a conspiracy theory about the Smoleńsk airplane crash to the ones who did not. They found that people endorsing a conspiratorial interpretation of the catastrophe preferred to distance themselves from skeptics, while skeptics preferred greater distance to conspiracy believers. This effect, however, was stronger in the group of skeptics than in the group of believers, probably because conspiracy beliefs are socially stigmatized. In addition, more global interpretation of national history played a role in this specific conspiratorial belief - people who believed that their group is unique in terms of historical suffering were more prone to interpret the event in a conspiratorial way. 

Taken together, findings from these two studies illustrate how the spread of conspiratorial thinking following a collective trauma in certain society can be detrimental to intergroup relations, but also how it contributes to intragroup divides as well.

Research conducted by **Lukić, Žeželj, and Stanković (2019, this issue)** explored a curious phenomenon of believing in mutually exclusive conspiracy theories. The authors replicated previous findings indicating the complexity of the conspiratorial pattern of thinking, i.e., the possibility to believe completely contradictory conspiracy beliefs at the same time. Additionally, their fine-grained analyses revealed mechanisms of the reasoning behind the endorsement of contradictory conspiracy theories. Namely, in overcoming contradictory beliefs people tend to use two strategies: first, the crucial content of the conspiracy theory is extracted while the other information is devalued; second, contradictory scenarios of the same event are treated as possible versions. In other words, these findings demonstrated that conspiracy theories believers are not as irrational as typically portrayed in contemporary literature.

The irrationality of irrational beliefs is further contested in the following two papers in which the authors examine the consequences of superstitious beliefs and beliefs in paranormal phenomena.

As opposed to the accumulated evidence that irrational beliefs lead to negative personal and societal outcomes, **Kostovičová (2019, this issue)** experimentally investigated a controversial claim that inducing a luck-related superstition, paradoxically, leads to more rational thinking, i.e. better performance on a list of cognitive tasks (CRT, conjunction fallacy, probabilistic reasoning). She found a negative effect of “lucky charm” on boys', but a positive effect on girls' performance; the effect in girls was mediated by increased self-efficacy. It implies that induction can be beneficial to certain groups, by lowering anxiety and increasing confidence, but detrimental to others.

Inspired by the persistence and widespreadness of so-called extra-sensory perception (ESP) beliefs, such as belief in the ability to communicate via thoughts, or in the ability to predict future events, **Branković (2019, this issue)** explored which basic motivations and cognitive styles are related to it. She found that more proneness to experiential thinking predicts more endorsement of ESP beliefs; as for motivational part, ESP beliefs were positively related to fear of death, and this relation was mediated by the belief that chance controls a person's life. These findings open door for future research about the protective function of such beliefs.

These two studies provide some evidence of potentially positive effects of endorsing irrational beliefs - through alleviating performance anxiety or even existential anxiety. The evidence should, however, be treated as preliminary, as the effects are small and context-dependent - therefore future direct and conceptual replications are warranted.

While Branković viewed experiential/rational cognitive styles as antecedents of ESP beliefs, **Jokić and Purić (2019, this issue)** investigated personality determinants of the two cognitive styles (Epstein, 1991; Epstein, Pacini, Denes-Raj, & Heier, 1996). The authors focused on a broader personality space, defined by the HEXACO model (Ashton & Lee, 2007) and Trait Emotional Intelligence (TEI - Petrides & Furnham, 2001). The results of this study revealed that four thinking styles can be defined by combining rational and experiential dimensions. Although experiential thinking leads to irrational and superstitious beliefs, when combined with high rationality, irrational beliefs can be replaced by rational and logical thinking. Out of the personality traits, TEI was the crucial predictor of experiential thinking and the second best predictor of rational thinking, after Openness to experiences.

Finally, **Erceg, Galić, and Bubić (2019, this issue)** did not focus on personality traits or motivations, but rather on cognitive and rational thinking abilities as determinants of a broader category of so-called “epistemically suspect beliefs”, that incorporate superstitious beliefs, belief in luck, paranormal and ESP beliefs. The authors argue that all these beliefs share one common feature, and that is ontological confusion - for example, blurring the ontological distinction between categories of phenomena, and quote empirical evidence of these beliefs form one higher-order factor (Lindeman & Aarnio, 2006; Lindeman & Svedholm, 2012). They found that rational thinking abilities, cognitive styles, and self-control, but not intelligence, significantly predicted the endorsement of epistemically suspect beliefs. Their results point to the fact that intelligence and rational thinking should not be equated, and that one can deviate from norms for rational reasoning independent of their intelligence.

Taken together, this collection of papers illustrates the diversity of contemporary research on irrational beliefs. Apart from the descriptive portion of the data, which has merit on its own, the authors provide important methodological innovations in the way these beliefs are measured, but also look deeper in their functionality and consequences. The reader should keep in mind that this special issue was meant not to provide an exhaustive overview of such a wide topic, but to familiarize them with a variety of methods, topics, and approaches. We hope that it would be thought-provoking, especially due to the fact that the authors restrain from value-judgments when it comes to people who endorse them and even explore the potential positive consequences of certain irrational beliefs.
